# Influenza SIRS with Minimal Pneumonitis

**DOI:** 10.3389/fmed.2016.00037

**Published:** 2016-08-31

**Authors:** Shruti Erramilli, Praveen Mannam, Constantine A. Manthous

**Affiliations:** ^1^Pulmonary, Critical Care and Sleep Medicine, Department of Internal Medicine, Yale University School of Medicine, New Haven, CT, USA; ^2^Lawrence and Memorial Hospital, New London, CT, USA

**Keywords:** systemic inflammatory response syndrome, influenza, ARDS, endothelium, neuraminidase inhibitors

## Abstract

Although systemic inflammatory response syndrome (SIRS) is a known complication of severe influenza pneumonia, it has been reported very rarely in patients with minimal parenchymal lung disease. We here report a case of severe SIRS, anasarca, and marked vascular phenomena with minimal or no pneumonitis. This case highlights that viruses, including influenza, may cause vascular dysregulation causing SIRS, even without substantial visceral organ involvement.

## Introduction

Influenza viruses are among the most common respiratory tract infections in humans and are a common cause of morbidity and mortality (1). Although systemic inflammatory response syndrome (SIRS) has been described after influenza infection, nearly all reported cases had severe lung injury and/or bacterial infections (2, 3). Here, we describe an unusual presentation of influenza infection with severe SIRS and multiple organ dysfunctions but without parenchymal infiltrates or lung injury.

## Case Presentation

**Figure 1:**
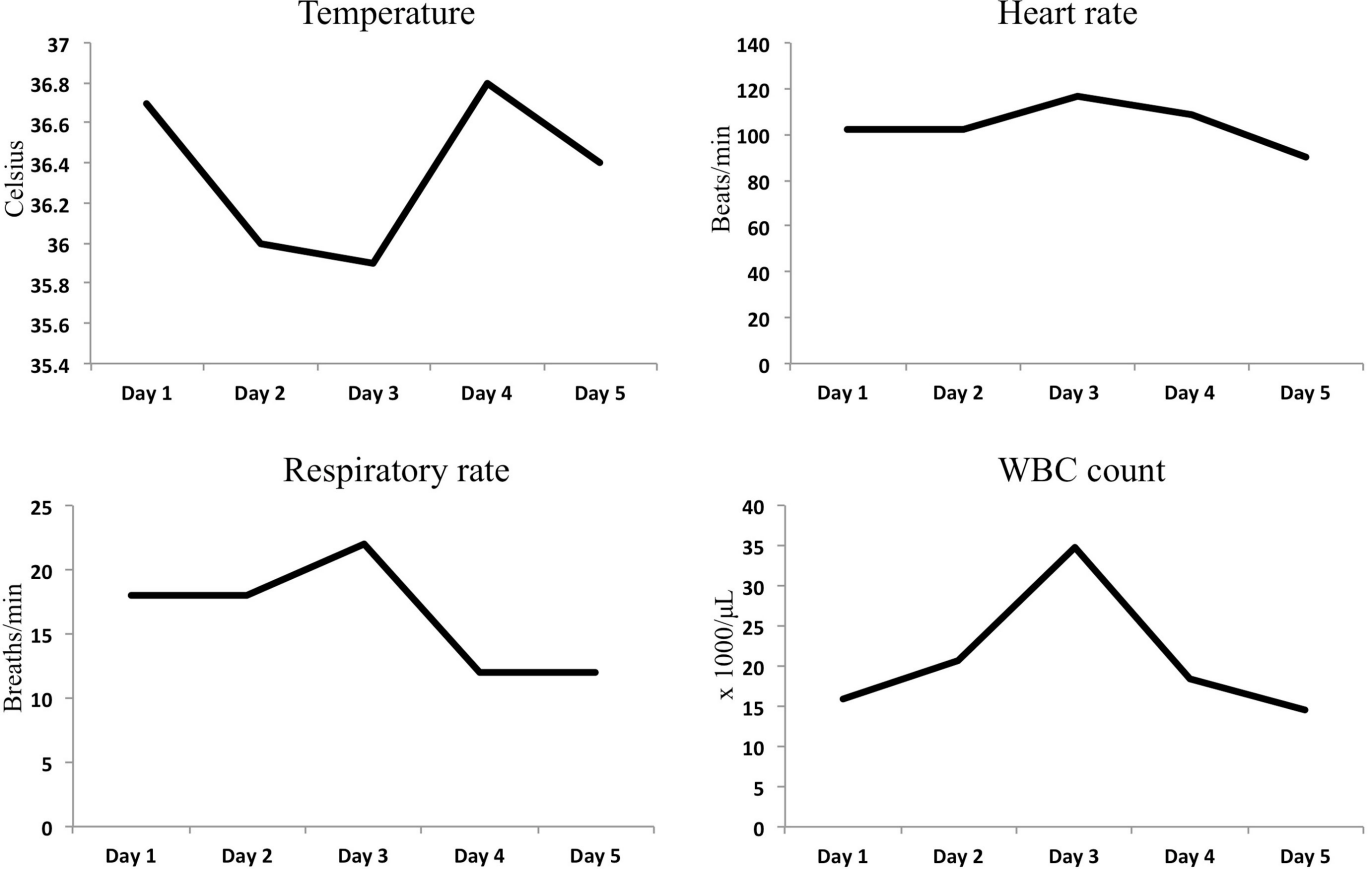
**Graphical representation of SIRS criteria trends during ICU stay**.

**Figure 2:**
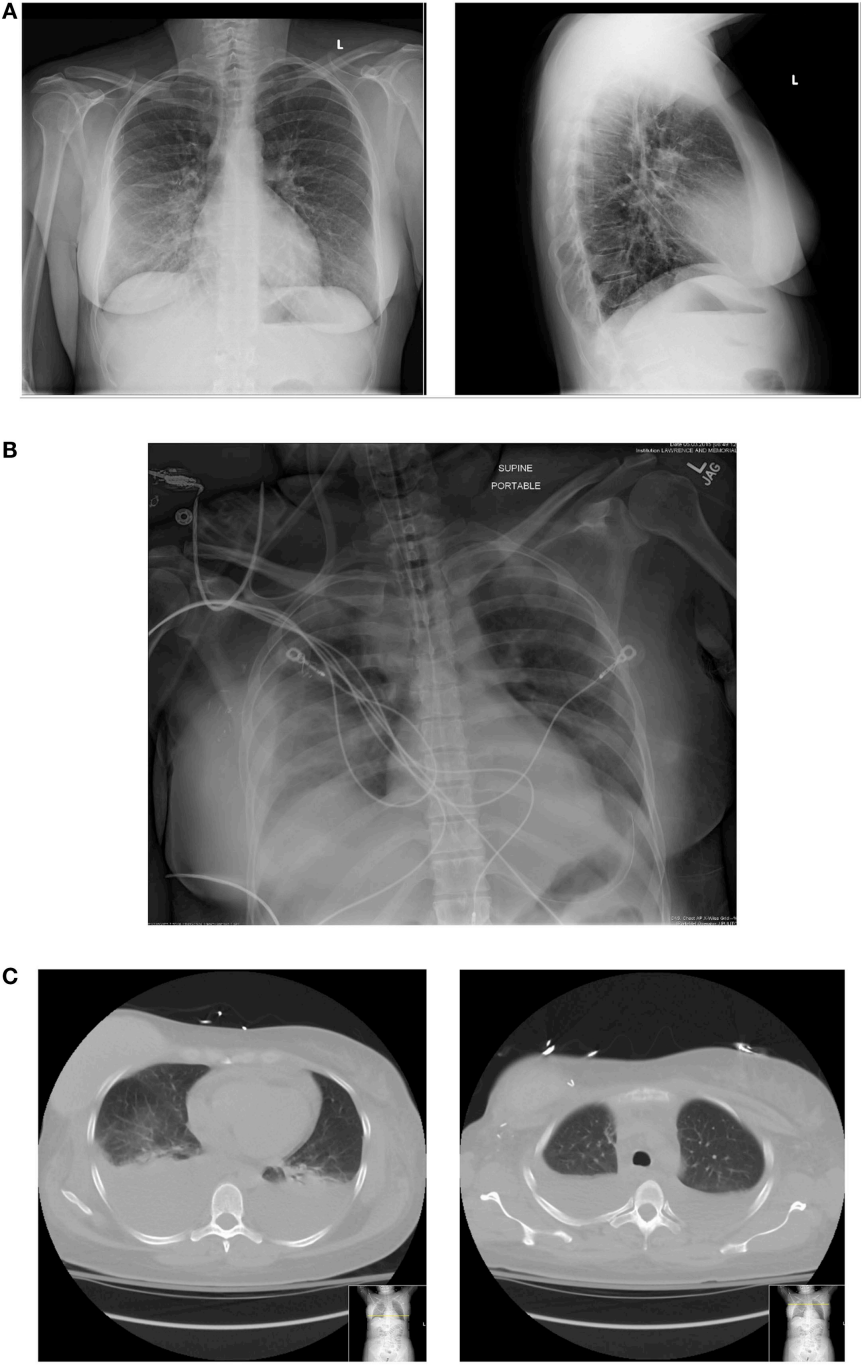
**(A)** Initial chest radiograph. **(B)** Chest radiograph 48 h at ICU admission. **(C)** Corresponding CT chest with representative cuts at bases (just above diaphragm) and upper-chest showing large pleural effusions and some adjacent compressive atelectasis but little/no parenchymal infiltrate.

A 44-year-old female with a remote past medical history of Stage IIb breast cancer, treated with mastectomy, chemotherapy, and radiation, presented with complaints of non-productive cough, malaise, and lower extremity paresthesias of 1 week duration. On presentation, her vitals were BP 110/62, heart rate 102, respiratory rate 18, and O_2_ saturations of 100% on room air (Figure 1). She was initially admitted to hospital wards, but became increasingly restless with leg pain, and developed hypotension with blood pressure of 80/50, leading to ICU transfer on the second hospital day. At the time of transfer, she was noted to be hypotensive with leukocytosis (WBC of 34.8 × 1000/μL), hemoconcentration (Hgb of 17.8 g/dL), thrombocytopenia (platelet count of 70 × 1000/μL), hyponatremia (Na+ of 129 mmol/L), and lactic acidosis (5.3 mmol/L). Creatine kinase was 966 U/L, and renal function and electrolytes were normal. She was hypoalbuminemic (1.6 g/dL), and her procalcitonin was (low) 0.25 μg/L. She had an elevated troponin I levels, 0.62 ng/mL (Ref range <0.06) and a normal EKG. Liver function tests showed mild elevation in AST 48 U/L (Ref range 15–37 U/L) and low alkaline phosphatase 37 U/L (Ref range 45–117 U/L). The bilirubin and AST levels were normal. Chest radiograph suggested a possible, subtle right lower lobe interstitial infiltrate (Figure 2). A rapid test nasal swab was positive for influenza B, and she was treated with oseltamivir and levofloxacin. Her hypotension became persistent and unresponsive (more than 7 L fluid administered on day of ICU transfer and 14 L over 48 h) prompting addition of norepinephrine and phenylephrine titrated to a systolic blood pressure of 100 mmHg. Antibiotics were also broadened to include vancomycin and piperacillin/tazobactam. Computed tomography of chest, abdomen, and pelvis demonstrated anasarca with bilateral pleural effusions, mild peri-effusion compressive atelectasis, and ascites. Lung parenchyma was otherwise clear (Figure 2). Her most remarkable complaint was severe lower extremity pain/paresthesias associated with profound (gray/purple) mottling. Screening for disseminated intravascular coagulation showed a prothrombin time 13.8 s (Ref range 9.6–11.6 s), partial thromboplastin time 42.8 s (Ref range 21.9–31.4 s), and fibrinogen 205.2 mg/dL (Ref range 217–425 mg/dL) but normal d-dimer. The ISTH DIC score was 4 suggesting absence of DIC. Lupus anticoagulants 1 and 2 were slightly above normal (50 and 36 s, upper limits 42 and 35 s). Ultrasounds of her extremities did not demonstrate thrombosis. Gram stain and cultures of blood, sputum, and urine showed no bacterial pathogens, and procalcitonin levels remained low on multiple serial tests. The coagulopathy reversed and she was weaned off vasopressors on day 3. She never developed respiratory failure, requiring modest supplemental oxygen of 2 L O_2_ throughout, and discharged from ICU on day 5.

## Discussion

Although there are many reports of SIRS associated with influenza, few if any cases have been described without primary lung involvement, i.e., influenza pneumonia/ARDS. While cases of primary influenza SIRS without pneumonia may be embedded in case series (4–6), we could find only two cases of influenza SIRS, explicitly described to have no pneumonia (7, 8).

The primary clinical manifestations of influenza infection are in the lung. The primary portal entry of influenza is the aero-digestive tract, specifically the respiratory epithelium. Epithelial infection causes upper respiratory infection, which may spread distally to cause tracheobronchitis. When small airways/airspaces become infected, in the most severe cases, it may cause distal inflammation, pulmonary edema, and ARDS. This may occur, in part, by impairing edema fluid clearance by inhibiting epithelial Na channel (ENaC) (9, 10). Influenza also stimulates inflammatory cells such as lung macrophages, neutrophils, and T cells that may contribute to “cytokine storm,” which may promote lung injury and SIRS (11, 12). Recent studies have demonstrated that endothelial cells play an important role in the pathogenesis of SIRS associated with influenza infection (13). Mechanisms of endothelial injury include cytokine-mediated disruption of cell–cell junctions. Activated endothelial cells may express adhesion molecules that recruit inflammatory cells such a neutrophils leading to further injury (14, 15). Influenza may also directly infect endothelial cells with activation of NF-κB causing apoptosis (16).

Our patient presented with non-productive cough and no interstitial parenchymal infiltrates on CT scan. She never developed severe pneumonia or ARDS, whereas influenza B manifested mainly as severe SIRS. We thus speculate that cytokine storm and endothelial dysfunction may have been the primary pathogenic mechanism of her illness. The presence of small vessel clinical signs (profound skin mottling), diffuse capillary leak (with large effusions, ascites, and anasarca), coagulation abnormalities, and lupus anticoagulant during our patient’s illness support this hypothesis. She developed no signs of macro-vascular thrombosis. Lupus anticoagulation has been reported to interact with the endothelium and impairs prostacyclin release to induce thrombosis (17–19). Our patient’s course is consistent with a growing literature describing endothelial dysfunction in viral infections (20). Evidence suggests that treatment with neuraminidase inhibitors reduces severe clinical outcomes in patients hospitalized with influenza. A review of the hospitalized patients with H1NI infection 2009–2010 showed that administration of neuraminidase inhibitors was associated with a reduction in mortality risk [adjusted odds ratio (OR) 0.81; 95% CI 0.70–0.93; *p* = 0.0024]. Although early initiation of treatment is advocated, there appears to be a benefit even among those starting treatment up to 5 days after symptom onset (21, 22). The side effects associated with treatment are typically mild such as transient upper respiratory and gastrointestinal symptoms (23). At present, there are no specific therapies to treat the extensive endothelial damage associated with infection except for supportive care.

It is possible that our patient had an occult, unidentified bacterial infection that responded to antibacterial therapy. Nonetheless, the weight of evidence, including a CT scan of chest, abdomen, pelvis without parenchymal infiltrates or any other source, negative cultures, no localizing signs of alternate infection, and low procalcitonin levels, suggests that our patient had only influenza infection. We believe this case highlights the growing appreciation that viruses can cause SIRS, and in the case of influenza, this may occur in the absence of pneumonia or severe lung injury.

The patient provided informed consent and IRB approval was not required by the institution for publication of the case report.

## Author Contributions

SE, PM and CM were involved in the clinical care of the patient. SE, PM, and CM did the background research, drafting of manuscripts, and final edits.

## Conflict of Interest Statement

The authors declare that the research was conducted in the absence of any commercial or financial relationships that could be construed as a potential conflict of interest.

## Supplementary Material

Table S1 **SIRS criteria values during ICU stay**.
