# Supplementary material for: Influenza SIRS with Minimal Pneumonitis
Source: Front Med (Lausanne). 2016 Aug 31;3:37. doi: 10.3389/fmed.2016.00037 (PMC5005447; doi:10.3389/fmed.2016.00037)
Supplement: Table S1 — SIRS criteria values during ICU stay. [file Table_1.DOCX]

Table 1

|  | Day 1 | Day 2 | Day 3 | Day 4 | Day 5 |
| --- | --- | --- | --- | --- | --- |
| Temperature (Celsius) | 36.7 | 36 | 35.9 | 36.8 | 36.4 |
| Heart rate (Beats/min) | 102 | 102 | 117 | 109 | 90 |
| Respiratory rate (Breaths/min) | 18 | 18 | 22 | 12 | 12 |
| WBC count (x 1000/μL) | 15.8 | 20.6 | 34.8 | 18.5 | 14.5 |
